# Exploring the impact of pharmacist comprehensive annual care plans on perceived quality of chronic illness care by patients in Alberta, Canada

**DOI:** 10.1177/17151635211020340

**Published:** 2021-07-05

**Authors:** Candace Necyk, Jeffrey A. Johnson, Ross T. Tsuyuki, Dean T. Eurich

**Affiliations:** School of Public Health, University of Alberta, Edmonton, Alberta; Faculty of Pharmacy and Pharmaceutical Sciences (Necyk), University of Alberta, Edmonton, Alberta; Faculty of Pharmacy and Pharmaceutical Sciences, University of Alberta, Edmonton, Alberta; Faculty of Medicine and Dentistry, University of Alberta, Edmonton, Alberta; Faculty of Pharmacy and Pharmaceutical Sciences, University of Alberta, Edmonton, Alberta

## Abstract

**Background:**

In 2012, the Government of Alberta introduced a funding program to remunerate pharmacists to develop a comprehensive annual care plan (CACP) for patients with complex needs. The objective of this study is to explore patients’ perceptions of the care they received through the pharmacist CACP program in Alberta.

**Methods:**

We invited 3442 patients who received a pharmacist-billed CACP within the previous 3 months and 6888 matched controls across Alberta to complete an online questionnaire. The questionnaire consisted of the short version Patient Assessment of Chronic Illness Care (PACIC-11), with 3 additional pharmacy-specific assessment questions added. Additional questions related to health status and demographics were also included.

**Results:**

Overall, most patients indicated a low level of chronic illness care by pharmacists, with few differences noted between CACP patients and non-CACP controls. Of note, controls reported higher quality of care for 5 domains within the adapted PACIC-like tool compared with CACP patients (*p* < 0.05 for all). Interestingly, only 79 (44%) of CACP patients reported that they had received a CACP, whereas only 192 (66%) of control patients reported that they did not receive a care plan. In a sensitivity analysis including only these respondents, individuals who received a CACP perceived a significantly higher quality of chronic illness care across all PACIC domains.

**Conclusion:**

Overall, chronic illness care incentivized by the pharmacist CACP program in Alberta is perceived to be moderate to low. When limited to respondents who explicitly recognized receiving the service or not, the perceptions of quality of care were more positive. This suggests that better implementation of CACP by pharmacists may be associated with improved quality of care and that some redesign is needed to engage patients more. *Can Pharm J (Ott)* 2021;154:xx-xx.

**Knowledge Into Practice**

- Various chronic disease management initiatives have been trialed around the world, demonstrating inconclusive results on their impact.
- A population-wide remuneration model for pharmacists to develop comprehensive annual care plans (CACPs) for patients with chronic disease is novel to Alberta, Canada, and its impact on patient perceptions of chronic illness care remains unknown.
- Results of this research are the first to quantitatively evaluate patient perspectives on chronic illness care related to a pharmacist-billed CACP. Our results demonstrate that having a pharmacist-billed CACP does not lead to substantially improved chronic illness care from a patient perspective.
- Our study highlights that a single annual CACP without a required and close follow-up of patients is unlikely to affect patient outcomes.
- A program design with more structure, treatment goals and that includes required follow-up visits is needed to better realize the full potential of this population-level intervention from a patient perspective.

**Mise En Pratique Des Connaissances**

- Diverses initiatives de gestion de maladies chroniques ont été testées partout dans le monde, avec des résultats peu concluants quant à leur impact.
- Un modèle de rémunération au niveau de la population pour que les pharmaciens élaborent des régimes de soins annuels complets pour les patients atteints de maladies chroniques est nouveau en Alberta, au Canada, et l’impact sur les perceptions des patients concernant les soins des maladies chroniques reste inconnu.
- Les résultats de cette recherche permettront d’évaluer quantitativement les points de vue des patients sur les soins des maladies chroniques liés à un régime de soins annuel facturé par un pharmacien. Nos résultats démontrent que le fait d’avoir un régime de soins annuel facturé par le pharmacien ne conduit pas à une amélioration substantielle des soins des maladies chroniques du point de vue du patient.
- Notre étude souligne qu’un seul régime de soins annuel sans le suivi nécessaire des patients est peu susceptible d’avoir un impact sur les résultats des patients.
- Une conception de programme plus structurée, avec des objectifs de traitement et des visites de suivi obligatoires, est nécessaire pour mieux réaliser le plein potentiel de cette intervention au niveau de la population du point de vue du patient.

## Introduction

Chronic diseases are defined as medical conditions that are often long term and do not have a cure (e.g., asthma).^1^ The prevalence of chronic diseases is increasing worldwide, and it is projected that by 2030, the number of deaths due to chronic disease will be 52 million.^2^ The consequences to an individual living with chronic disease are extensive, including impaired mobility, inability to work, increased physician and hospital visits, increased prescription medications and even early death.^1^ Given the prevalence, cost to the health care system for the management of chronic diseases is significant; it is reported that individuals living with chronic diseases account for more than two-thirds of hospital admissions, more than one-third of all physician visits and more than one-quarter of emergency department (ED) visits.^1^ More than 735,000 people in Alberta were identified as living with hypertension, diabetes, chronic obstructive pulmonary disease and coronary artery disease in the fiscal year 2012-2013, costing the Alberta government more than $4.5 billion in 1 year for the management of these 4 chronic diseases.^1^ In 2014, the Auditor General of Alberta released a report strategizing the immediate need for improved chronic disease management (CDM).^1^

Patient engagement is identified as a key factor in effective CDM, since patients spend most of their time outside of physician offices and need to self-manage their own conditions.^1^ Patient engagement in their CDM has also been linked to improved health outcomes.^3^ The development of a care plan is a cornerstone in CDM, since it links the services provided by the health care professional to the self-management expectations required by the patient.^1^ Such a model is congruent with the chronic care model, which has demonstrated improved health outcomes in patients who are informed and activated in their CDM, occurring alongside other interdependent factors such as availability to community resources.^4-6^ Specifically, it was designed to measure patient activation, goal setting, problem solving, delivery system design/decision support and follow-up/coordination.^4^

In 2012, Alberta Health, the provincial health ministry, introduced a CDM initiative in the form of Comprehensive Annual Care Plans (CACP) by pharmacists.^7^ Given pharmacists’ rapidly growing scope of practice in Alberta, including ordering laboratory tests and initial access prescribing, pharmacists are in an ideal position to help patients manage their chronic diseases.^8^ CACPs are intended to be developed in collaboration with the patient and outline the patient’s medical history, medications and specific goals and timelines of further medical and laboratory tests and nonpharmacologic therapy such as exercise; pharmacist CACPs should place a particular focus on drug therapy needed to manage a patient’s chronic disease.^1^ The CACP should reflect the patient’s values and personal health goals as they relate to his or her complex health care needs.^1^ Pharmacists in Alberta are remunerated by Alberta Health once annually for the preparation of a CACP for patients with 2 or more of the following conditions: asthma, diabetes, chronic obstructive pulmonary disease, heart disease, heart failure, hypertension or mental health disorders.^7^ Recognizing that some patients may receive similar care plans for which their pharmacist does not submit a billing claim to Alberta Health, we use the term *pharmacist-billed CACP* to indicate those that have been remunerated by Alberta Health.

To date, little evaluation has been undertaken on the effectiveness of pharmacist-billed CACPs in enhancing CDM. Given the important role of the patient in their own CDM, the patient experience with their disease management is an important criterion to evaluate when analyzing the overall effectiveness of CACPs. Other researchers qualitatively explored pharmacist CACPs in Alberta, and their results suggest that some patients have positive perceptions regarding the knowledge and support for meeting their health goals gained through a CACP as well as improved access to care.^8,9^ This study was limited, however, by the lack of comparison of perceptions of patients who did not receive a CACP. Therefore, the objective of this current study is to assess patients’ perspectives of the pharmacist CACP on their chronic illness care. We hypothesized that patients who receive a pharmacist-billed CACP in Alberta will have better perceived quality of chronic illness care compared with those patients who do not receive a CACP.

## Methods

A cross-sectional survey design was used to assess Alberta patients’ perceived quality of chronic illness care associated with CACPs. Between February and December 2019, individuals older than 18 years of age who received pharmacist-billed CACP in the previous 3 months were identified by Alberta Health, based on billing codes.^7^ In addition, for each person who received a CACP, up to 2 matched controls with similar age (within 5 years), sex, qualifying CACP conditions, service provider (i.e., same pharmacy) and date of service (within 1 year), but who did not receive a pharmacist-billed CACP, were also invited to participate to establish a control group for the study. Only the first eligible CACP (or the pseudo-index CACP date in the control group, based on the matched case’s index date) was used to identify potential participants; once a participant was contacted, they were excluded from further survey distributions to prevent repeated data from the same participant.

Cover letters containing relevant study information and a unique URL to access the online survey for either the CACP group or the control group were mailed out to all selected participants in both groups. The selection and mail-out process occurred 3 times (February 2019, June 2019 and December 2019) until our required sample size was obtained. Survey responses were collected in Qualtrics XM platform. All participant identification, matching and mail distribution were completed by Alberta Health so as to maintain the confidentiality of personal health information of the participants.

The study received approval from the University of Alberta Human Research and Ethics Board.

### Survey measures

The primary outcome measure for this evaluation was the 11-item version of the Patient Assessment of Care for Chronic Conditions (PACIC) questionnaire.^10^ The PACIC was developed to assess the degree to which the care provided to patients is congruent with the CCM, from the perspective of the patient.^11^ The questionnaire asks patients the extent to which they have received specific care and actions related to chronic disease care from their health professional (in this case, their pharmacist), with responses ranging from 0% to 100%. Responses have been categorized as low (0-30%), medium (40%-60%) and high (70%-100%)^10^; since the tool built into our questionnaire allowed for selection by 1 unit, or percentage, by the respondent, we modified the categories to 0% to 39% = low, 40% to 69% = medium and 70% to 100% = high and opted to adapt the word *medium* to *moderate*. The 11-item PACIC has been validated in patients with diabetes, in whom the PACIC total score is associated with increased physical activity, receiving appropriate laboratory assessments and self-management counselling.^12^ The longer 20-item version of the PACIC has been widely used in research across the majority of other chronic diseases as well.^13-16^ Based on the expertise of our steering committee, we added 3 questions, with similar format, with a particular focus on the pharmacy practice setting and the collaborative intention of CACPs. The additional questions were: “Told how visits with other types of health professionals would help your treatment,” “Given enough time to talk about your medical conditions or medications” and “Told your pharmacists would work together with other health professionals to coordinate your care.”

Based on our team’s previous experience using the PACIC-11,^17,18^ we anticipated the SD of the total score to be approximately 25. As there is no prespecified minimally important difference for PACIC scores, we estimated an effect size of 0.3, or an absolute difference of 7.5 points, to be clinically important. To observe such a difference/effect size with 80% power and a probability of type 1 error of 5%, we required 131 patients who received a CACP and 262 controls (393 total). We initially anticipated a 35% response rate for the survey (based on recent population surveys undertaken by Alberta Health), resulting in the need to contact 1123 selected patients (i.e., 374 exposed and 749 unexposed). Additional mailouts were sent out to meet our prespecified sample size.

Immediately following the PACIC questions in the survey, respondents were asked to rate their overall satisfaction with care they receive by their pharmacy using a 6-point Likert-type scale, with 1 = *very dissatisfied* and 6 = *very satisfied*. Secondary outcome measures in the survey included the 5-level EQ-5D, including the visual analogue scale (VAS) self-rating of health.^19,20^ The Patient Health Questionnaire–2 (PHQ-2) and Generalized Anxiety Disorder Scale–2 (GAD-2) were used to evaluate the mental health of the respondents; scores of 3 and greater on both scales are considered highly specific and sensitive for the positive screening of each disorder.^19-22^ To explore the potential impact of health literacy on our results, a Single Item Literacy Screener was included.^23^

Lastly, 2 questions were included at the beginning of the survey to explore whether respondents who received a pharmacist-billed CACP were aware that they had received one and whether they were asked to sign the document, as this is a preestablished expectation of the CACP remuneration model; in the control group, these questions serve to explore whether they were aware they had not received this service. The 2 questions were, “In the last 3 months, did you spend time with your pharmacist to review your medical conditions in order to create a detailed treatment plan?” and “In the last 3 months, do you recall signing a treatment plan or medication review at your pharmacy?”; the respondent was asked to respond yes or no to both questions. Questions related to sociodemographic characteristics (age, sex, marital status, education level, annual income, ethnicity) and qualifying chronic disease status were included at the end of the survey to fully summarize the cohort of respondents, for possible adjustment for unbalanced characteristics in our analyses if necessary. A copy of the survey is provided in Appendix 1, available in the online version of the article.

### Statistical Analysis

Proportions were calculated for all categorized variables describing the demographics of the study population. Proportions and 95% confidence intervals (CIs) were calculated for the PHQ-2 and GAD-2, categorized into proportions of individuals reporting a score of 3 and greater and less than 3, as well as those reporting they received a care plan and signed a care plan. Means and standard deviations were calculated for age, VAS score, satisfaction of care from the pharmacist and Single Item Literacy Screener. The Canadian EQ-5D-5L value set was used to generate an index value and standard deviation for the EQ-5D-5L in each study group.^20^ Only 34% of the total study population responded to all 14 PACIC questions, our main outcome measure. Give the high nonresponse rate, a total PACIC-11 score or mean of all 14 PACIC-like questions on the survey was not computed, since complete case analysis would substantially reduce the sample size of our study population (34% of the CACP patients and 36% of the controls). As such, one-way analysis of variance tests were used to determine the association between responses to each of the PACIC items according to CACP group. All analyses were completed using Stata 14 (StataCorp, Stata Statistical Software, release 14, College Station, TX).

### Sensitivity Analysis

A post hoc subgroup analysis was undertaken to evaluate the PACIC outcomes in those patients who received a pharmacist-billed CACP and answered “yes” to the question, “In the last 3 months, did you spend time with your pharmacist to review your medical conditions in order to create a detailed treatment plan?” and the subset of the control group who did not receive a CACP and answered “no” to the above question. The purpose of the sensitivity analysis was to further assess a subgroup of patients within our cohorts who were aware and likely engaged in the CACP development process with their pharmacist and to eliminate those who may have received a CACP but did not have it billed through Alberta Health or who were engaged in another type of medication review with the pharmacist that could be perceived as a CACP.

## Results

A total of 3500 CACP patients and 7000 controls were invited to participate in the study. After eliminating letters that were returned to sender, the final sample frame was reduced to 3442 CACP patients and 6888 control patients. A total of 178 CACP patients and 341 control patients participated in the online survey, demonstrating a response rate of 5.2% and 5%, respectively. Respondents in both the CACP and control cohorts had a mean age of 64 years, with approximately 46% female (Table 1). Marital status, highest level of education and annual income were similarly distributed across the 2 groups, with most reporting a marital status of married/common law (>60%), completed college or technical school (>30%) and an annual income of $50,000-99,999 (>30%). Ethnicity was similar across CACP patients and controls, with the majority being Caucasian (>80%). The proportion of qualifying CACP conditions between the 2 groups was similar, with hypertensive disease and diabetes mellitus being the most commonly reported. The health literacy abilities were similar between both groups (1.5 [SD 0.9] vs 1.7 [SD 1.1] in CACP patients vs controls; *p* = 0.07).

**Table 1:** Demographics of the study population

Characteristic (number of respondents in the CACP group; number of respondents in the control group)	CACP group (*n* = 178)	Control group (*n* = 341)	*p* value
Age (*n* = 147; 273) mean, SD	64 (12)	64 (12)	0.73
Sex (*n* = 162; 306), %			0.96
Female	46	46	
Male	54	54	
Marital status (*n* = 162; 306), %			0.83
Single/never married	10	12	
Married/common law	69	63	
Separated/Divorced	9	12	
Widowed	11	11	
Prefer not to respond	2	2	
Education level (*n* = 162; 306), %			0.59
Less than high school	7	6	
High school	27	26	
College/technical school	35	36	
Postsecondary	16	18	
Postgraduate	13	11	
Prefer not to respond	1	2	
Annual income (*n* = 162; 303), %			0.30
<$20,000	7	10	
$20,000–$49,999	20	24	
$50,000–$99,999	33	30	
>$100,000	24	19	
Prefer not to respond	17	18	
Ethnicity (*n* = 162; 304), %			0.18
Caucasian	83	88	
Aboriginal/Indigenous	1	1	
African	0.6	0.7	
Hispanic/Latino	0.6	0.3	
Caribbean	0.6	0.3	
East Asian	1	2	
South Asian	4	3	
Middle Eastern	0	0.3	
Prefer not to respond	4	1	
Qualifying conditions (*n* = 128; 248), %			
Asthma	20	23	0.50
Chronic obstructive pulmonary disease	13	12	0.74
Ischemic heart disease	9	4	0.09
Hypertensive disease	54	44	0.07
Heart failure	10	8	0.62
Diabetes mellitus	39	40	0.93
Mental health disorder	22	21	0.77

CACP, comprehensive annual care plan.

The general health status of the respondents, as measured by the EQ-5D-5L Index score and VAS, did not differ significantly between the 2 groups (Table 2). With regard to mental health status, the proportion of patients with a PHQ-2 score of 3 or greater was 16% and 18% (p = 0.62) in the CACP group and control group, respectively, and the proportion of patients with a GAD-2 score of 3 or greater was 15% and 18% (*p* = 0.39) in the CACP group and control group, respectively.

**Table 2:** Health and literacy status of study population

Survey question (number of respondents in the CACP group; number of respondents in the control group)	CACP group (*n* = 178)	Control group (*n* = 341)	*p* value
EQ-5D-5L index* (*n* = 160; 302)	0.79 (0.17)	0.76 (0.18)	0.23
EQ-5D-5L visual analogue scale* (*n* = 161; 308)	68 (19)	69 (19)	0.87
Single Item Literacy Screener* (*n* = 162; 306)	1.5 (0.89)	1.7 (1.1)	0.07
PHQ-2 score^†^ (*n* = 158; 300) ≥3	16% (11%–22%)	18% (14%–22%)	0.62
GAD-2 score^†^ (*n* = 158; 299) ≥3	15% (10%–21%)	18% (14%–23%)	0.39

CACP, comprehensive annual care plan; GAD-2, Generalized Anxiety Disorder Scale–2; PHQ-2, Patient Health Questionnaire–2.

* Mean (SD).

† Proportions (95% CI).

The response rates to all 14 PACIC-like questions ranged from a high of 80% to a low of 42% (Table 3). Importantly, the response rates for each question did not differ significantly between the CACP and control groups. In general, patients who receive a pharmacist-billed CACP reported similar average PACIC scores for each question as compared with controls (Table 3). Although some questions were rated moderately high in both groups (e.g., “satisfied that your care was well organized” (mean score 67 and 70 in those who received a CACP and controls, respectively; *p* = 0.33) a number of questions were rated low. Indeed, of the 11 validated PACIC questions, both CACP patients and controls rated 9 (82%) of 11 questions below a mean score of 40. Moreover, statistical differences were noted in 4 questions: “Given choices about treatment to think about” (38 vs 29; *p* = 0.046), “Contacted after a visit to see how things were going” (30 vs 20; *p* = 0.046), “Given enough time to talk about your medical conditions or medications” (67 vs 59; *p* = 0.04) and “Told how visits with other types of health professionals would help your treatment” (27 vs 15; *p* = 0.01), whereby mean scores were higher in controls relative to CACP patients, respectively; although the clinical importance of these differences is uncertain. With respect to the 3 additional PACIC-like questions, only “given enough time to talk about your medical conditions or medications” was moderately high for either the CACP or control patients. Collectively, although some differences were noted, the CACP and control group were remarkably similar in their PACIC responses, suggesting low to moderate overall perceived care of their chronic illnesses regardless of CACP.

**Table 3:** PACIC scores in total study population

PACIC-11 survey questions (number of respondents in the CACP group; respondents in the control group)	Mean (SD)		*p* value
	CACP group (*n* = 178)	Control group (*n* = 341)	
Given choices about treatment to think about (*n* = 119; 224)	29 (35)	38 (38)	0.046
Satisfied that your care was well organized (*n* = 142; 285)	67 (36)	70 (35)	0.33
Helped to set specific goals to improve your eating or exercise (*n* = 91; 198)	28 (36)	34 (39)	0.21
Given a copy of your treatment plan (*n* = 91; 185)	38 (42)	36 (43)	0.68
Encouraged to go to a specific group or class to help you cope with your chronic condition (*n* = 79; 155)	17 (32)	17 (31)	0.99
Asked questions, either directly or on a survey, about your health habits (*n* = 102; 193)	37 (39)	40 (40)	0.53
Helped to make a treatment plan that you could carry out in your daily life (*n* = 80; 173)	27 (37	32 (39)	0.34
Helped to plan ahead so you could take care of your condition even in hard times (*n* = 78; 173)	23 (34)	32 (40)	0.06
Asked how your chronic condition affects your life (*n* = 84; 182)	32 (39)	39 (41)	0.16
Contacted after a visit to see how things were going (*n* = 75; 162)	20 (35)	30 (39)	0.046
Told how visits with other types of doctors, like an eye doctor or surgeon, would help your treatment (*n* = 75; 156)	18 (33)	23 (34)	0.28
**Additional pharmacy-specific survey questions (number of respondents in the CACP group; respondents in the control group)**			
Told how visits with other types of health professionals would help your treatment (*n* = 75; 158)	15 (30)	27 (36)	0.01
Given enough time to talk about your medical conditions or medications (*n* = 121; 232)	59 (40)	67 (38)	0.04
Told your pharmacist would work together with other health professionals to coordinate your care (*n* = 90; 178)	34 (40)	45 (44)	0.05

CACP, comprehensive annual care plan; PACIC, Patient Assessment of Chronic Illness Care.

Despite their responses to the PACIC, overall satisfaction with care or how care was organized by the pharmacist was relatively high, irrespective of the CACP group. Indeed, those individuals who did not receive a pharmacist-billed CACP reported a slightly higher level of satisfaction of care by their pharmacy compared with those who did receive a CACP (5.0 [1.3] vs 4.7 [1.4], respectively; *p* = 0.01; Table 4). Moreover, within the PACIC tool, CACP patients and controls reported similarly high satisfaction that their care was well organized (67 vs 70, respectively, *p* = 0.33). However, only 44% of CACP patients reported receiving a care or treatment plan by their pharmacist; interestingly, 44% of control patients also reported receiving a care plan from their pharmacist, despite never having a CACP billed through Alberta Health at the time of the survey distribution. When asked if they recalled signing a treatment plan from their pharmacist, this proportion dropped to 32% of CACP patients and, again, 30% of control patients reported signing a care plan from their pharmacist.

**Table 4:** Satisfaction of pharmacy care and care plan awareness of study population

Survey question (number of respondents in the CACP group; respondents in the control group)	CACP group **(*n* = 178)**	Control group **(*n* = 341)**	*p* value
Satisfaction with care from pharmacist* (*n* = 159; 308)	4.7 (1.4)	5.0 (1.3)	0.01
Participants reporting they received a care plan^†^ (*n* = 178; 340)	44% (37%–52%)	44% (39%–49%)	0.90
Participants reporting they signed a care plan^†^ (*n* = 174; 336)	32% (26%–40%)	30% (25%–35%)	0.62

CACP, comprehensive annual care plan.

* Mean (SD).

† Proportions (95% CI).

### Sensitivity analysis

In total, 79 CACP patients and 192 control patients were included in the sensitivity analysis. Demographics and overall health status in this subgroup did not differ significantly from the overall study sample (Appendix 2, available in the online version of the article). When evaluating those patients who had a pharmacist-billed CACP who reported receiving a care plan and those control patients who reported not receiving a care plan, the PACIC mean scores differed markedly from those reported in the overall study sample (Table 5). Specifically, patients who received a pharmacist-billed CACP reported significantly higher PACIC scores than the control group across all questions. The questions that differed most dramatically included “given a copy of your treatment plan” (mean score 56 vs 22, respectively; *p* < 0.001), “asked questions, either directly or on a survey, about your health habits” (mean score 59 vs 24, respectively; *p* < 0.001), “helped to make a treatment plan that you could carryout in your daily life” (mean score 46 vs 10, respectively; *p* < 0.001) and “asked how your chronic condition affects your life” (mean score 51 vs 17, respectively; *p* < 0.001). However, despite the higher scores, overall perceived chronic illness care across most of the questions remained low to moderately low, with few questions with moderate to high average scores.

**Table 5:** PACIC scores in the sensitivity analysis subgroup

PACIC-11 survey questions* (number of respondents in the CACP group; respondents in the control group)	CACP group (*n* = 79)	Control group (*n* = 192)	*p* value
Given choices about treatment to think about (*n* = 63; 116)	40 (37)	23 (32)	<0.001
Satisfied that your care was well organized (*n* = 70; 152)	76 (30)	61 (37)	<0.001
Helped to set specific goals to improve your eating or exercise (*n* = 48; 98)	42 (40)	19 (32)	<0.001
Given a copy of your treatment plan (*n* = 45; 94)	56 (40)	22 (37)	<0.001
Encouraged to go to a specific group or class to help you cope with your chronic condition (*n* = 35; 85)	29 (39)	8 (21)	<0.001
Asked questions, either directly or on a survey, about your health habits (*n* = 56; 96)	59 (38)	24 (33)	<0.001
Helped to make a treatment plan that you could carryout in your daily life (*n* = 41; 84)	46 (41)	10 (21)	<0.001
Helped to plan ahead so you could take care of your condition even in hard times (*n* = 37; 85)	37 (39)	16 (30)	<0.01
Asked how your chronic condition affects your life (*n* = 42; 87)	51 (42)	17 (31)	<0.001
Contacted after a visit to see how things were going (*n* = 35; 84)	38 (44)	13 (28)	<0.001
Told how visits with other types of doctors, like an eye doctor or surgeon, would help your treatment (*n* = 38; 82)	31 (40)	10 (23)	<0.001
**Additional pharmacy-specific survey questions (number of respondents in the CACP group; respondents in the control group)**			
Told how visits with other types of health professionals would help your treatment (*n* = 35; 82)	26 (37)	12 (26)	0.03
Given enough time to talk about your medical conditions or medications (*n* = 63; 116)	72 (35)	53 (41)	<0.01
Told your pharmacist would work together with other health professionals to coordinate your care (*n* = 44; 86)	53 (42)	24 (37)	<0.001

CACP, comprehensive annual care plan; PACIC, Patient Assessment of Chronic Illness Care. Sensitivity cohort: The CACP cohort includes individuals who were billed for a CACP by a pharmacist and answered “yes” to the question “In the last 3 months, did you spend time with your pharmacist to review your medical conditions in order to create a detailed treatment plan?” Control cohort includes individuals who were not billed for a CACP and answered “no” to the question “In the last 3 months, did you spend time with your pharmacist to review your medical conditions in order to create a detailed treatment plan?”

* Mean (SD).

## Discussion

Given the significant role that patients must play in managing their own chronic illnesses, such as diet, exercise and managing medications and monitoring practices, understanding the effect of the CACP initiative from the patient perspective is crucial. Overall, our results demonstrate that having a pharmacist-billed CACP does not lead to substantially improved chronic illness care from a patient perspective. Our sensitivity analysis suggests that having a more engaged patient in the care plan development process does markedly improve the perception of chronic illness care by the patient. This is not surprising given that an informed, activated patient is an important aspect of the CCM that leads to improved patient outcomes.^4^ It may also signal an overall lack of patients’ understanding of the goals of the CACP program, with poor explanation of the program provided by Alberta Health and the pharmacists providing the service.

### Comparison with other literature

Because Alberta is the only jurisdiction to introduce a population-wide fee-for-service model such as the CACP program for pharmacists, our study is the first to quantitatively evaluate the patient perspective on chronic illness care in such a setting. Most research exploring care plan development for patients with complex needs has focused on primary care physicians. Indeed, when evaluating remuneration models for primary care physicians, studies have shown a minimal impact on quality of health care delivered.^24–26^ However, many indicators of quality in these studies rely on the delivery of specific services and do not seek to gain a patient perspective of their care. Campbell et al. sought to evaluate the patient experience related to health care reform under the United Kingdom Quality and Outcomes Framework introduced in 2004, in which primary care physicians are remunerated for the development of care plans for patients with chronic disease.^27,28^ Unfortunately, patients actually reported less continuity of care and lower satisfaction of care under this model.^27,28^ In our study, the overall study population reported similar findings, in which those who received a pharmacist-billed CACP did not perceive a substantially higher level of care than the controls, as hypothesized.

Pharmacists have most often been and continue to be involved in clinical services related to medication management services, such as medication reviews.^29^ Although an improvement in outcomes specific to certain chronic diseases such as diabetes and hypertension tends to be demonstrated in a controlled research setting (i.e., randomized controlled trials), similar outcomes rarely translate to the real world.^30^ This is often because of a lack of expectations around regular pharmacist follow-up, unclear patient eligibility criteria, lack of program structure and poor program evaluation.^30^ This has been demonstrated in both British Columbia and Ontario for their population-wide medication review service provided by pharmacists.^30^ In fact, people were less likely to be offered a medication review through the Ontario MedsCheck program if they were taking a higher number of medications.^31^ Our research highlights similarities to these studies, in which a population-wide service model found minimal impact perceived at the patient level. Patient engagement in such initiatives may improve perceived care.

In a recent qualitative assessment of Alberta’s pharmacist CACP program, Schindel et al. found that while not all patients walked away from a pharmacist-billed CACP with a clear understanding of their treatment goals, those who did were more motivated to play a role in the care of their chronic illness.^9^ Further, Hughes et al. demonstrated patient perceptions of gained knowledge about medical conditions and medications as well as encouragement and support to reach health goals after receiving a CACP from a pharmacist.^8^ However, the reasons why a pharmacist would select a particular patient to receive a CACP in this research setting and why patients would accept or reject the service are unknown and may affect the extent of the perceived benefits found. Nevertheless, in our sensitivity analysis, we showed that when the patient actually recalls receiving a CACP (perhaps a marker of better engagement by the pharmacist), patient satisfaction is greater as compared with those who did not receive a CACP. It is also possible that the CACP program design was too vague in terms of its goals and expectations for what a pharmacist should do. In the minds of some pharmacists, a CACP might just be a thorough medication review (which is an important starting point but unlikely to change outcomes). Finally, the design of the CACP program was vague: there were no expectations to improve evidence-based therapies (e.g., to reach blood pressure targets, or ensure patients with heart failure were receiving a renin-angiotensin system inhibitor).

### Strengths and limitations

A strength of this study is the use of a random sample of all patients who received a pharmacist-billed CACP in Alberta in the previous 3 months, thereby reducing the potential biases such as pharmacist level of care and patient selection and involvement that may be present in a more controlled research setting. To further strengthen our data, 2 control patients for every CACP patient were included and closely matched on important criteria that might otherwise confound the data, such as age, sex, pharmacy provider and qualifying conditions for a CACP. The use of a control group helps tease out a possible CACP effect from the usual care that patients already receive from pharmacists in Alberta (indeed, some “control” patients might have received a CACP from their pharmacists that was not billed and therefore would not be captured). Lastly, our sensitivity analysis allowed us to explore a group of patients who were likely more aware and engaged in the CACP process.

It is important to also note the limitations of this study. First, a cross-sectional study design cannot infer causality; our study results are only a snapshot in time of the perceived chronic illness care of patients and matched controls after a CACP, or lack thereof, was delivered. The response rate to our survey was also quite low (~5%), therefore limiting generalizability to the entire Alberta population. The online URL provided in a letter may have added an extra barrier to accessing the survey but was necessary to maintain patient confidentiality. In addition, different biases are inherent to survey research. First, patients who responded to the survey may differ fundamentally from those who did not. Since participants had to access the survey online, this may have limited responses from those who did not have access to the Internet. The letters were also written in English, potentially reducing the participation of those who were unable to read English. Recall bias may further confound our results, since we are relying on patients recalling specific information about their interaction with a pharmacist up to 3 months prior. However, the PACIC-11 is a validated self-report tool for evaluating chronic illness care occurring up to 6 months prior.^10^ Unfortunately, missing responses for items within the PACIC-11 limited the available data for analysis and suggests the instrument may not resonate with patients in this context. Some other caveats include: we were unable to evaluate the quality of the developed CACPs and how this relates to perceived patient impact, it may be that the CACP program design is not specific enough or the expectations are too vague as to encourage guidelines-based care and it is also possible that patients do not realize what their pharmacist is doing for them (perhaps pharmacists are underselling themselves). Previous research suggests that patients are not aware of care planning services by pharmacists and that terms often used by pharmacists to describe a care plan, such as “medication review,” can blur patient awareness of the service they are receiving.^8,9,32^ We also could not account for patient follow-up specifically in our analyses; as such, it is a review of a single event (a CACP), when chronic disease care is a longitudinal phenomenon. However, the PACIC-11 asks patients to recall all visits with their pharmacist in the previous 3 months; therefore, follow-up visits may be reflected in their responses but cannot be quantitatively confirmed in our analyses.

## Conclusion

Overall, the chronic illness care provided by pharmacists in Alberta was perceived by patients to be moderate to low, irrespective of whether a patient received a pharmacist-billed CACP or not. Sensitivity analyses suggest a benefit of a pharmacist-billed CACP relative to controls who do not receive care plans. Patients’ perceptions of their chronic illness care suggest that the CACP program needs to be improved, perhaps with patients involved in the redesign. ■

## Supplemental Material

sj-pdf-1-cph-10.1177_17151635211020340 – Supplemental material for Exploring the impact of pharmacist comprehensive annual care plans on perceived quality of chronic illness care by patients in Alberta, Canada Click here for additional data file. Supplemental material, sj-pdf-1-cph-10.1177_17151635211020340 for Exploring the impact of pharmacist comprehensive annual care plans on perceived quality of chronic illness care by patients in Alberta, Canada by Candace Necyk, Jeffrey A. Johnson, Ross T. Tsuyuki and Dean T. Eurich in Canadian Pharmacists Journal / Revue des Pharmaciens du Canada

sj-pdf-2-cph-10.1177_17151635211020340 – Supplemental material for Exploring the impact of pharmacist comprehensive annual care plans on perceived quality of chronic illness care by patients in Alberta, Canada Click here for additional data file. Supplemental material, sj-pdf-2-cph-10.1177_17151635211020340 for Exploring the impact of pharmacist comprehensive annual care plans on perceived quality of chronic illness care by patients in Alberta, Canada by Candace Necyk, Jeffrey A. Johnson, Ross T. Tsuyuki and Dean T. Eurich in Canadian Pharmacists Journal / Revue des Pharmaciens du Canada
