# Supplementary material for: Exploring the impact of pharmacist comprehensive annual care plans on perceived quality of chronic illness care by patients in Alberta, Canada
Source: Can Pharm J (Ott). 2021 Jul 5;154(5):331–41. doi: 10.1177/17151635211020340 (PMC8408906; doi:10.1177/17151635211020340)
Supplement: sj-pdf-1-cph-10.1177_17151635211020340 – Supplemental material for Exploring the impact of pharmacist comprehensive annual care plans on perceived quality of chronic illness care by patients in Alberta, Canada [file sj-pdf-1-cph-10.1177_17151635211020340.pdf]

APPENDIX 1 Patient Questionnaire

You have been invited to participate in a research study on chronic disease care being conducted by investigators from the School of Public Health at the University of Alberta.

Study Title: *Exploring the Impact of Pharmacist Comprehensive Annual Care Plans on Perceived Quality of Care by Patients in Alberta*

For this project, you are asked to complete a survey, providing information about yourself, including how you rate your physical and mental health and your recent experiences with health services in Alberta. This brief survey should take about 10-15 minutes to complete.

Thank you.

Q1 In the last 3 months, did you spend time with your pharmacist to review your medical conditions in order to create a detailed treatment plan?

○ Yes

○ No

Q2 In the last 3 months, do you recall signing a treatment plan at your pharmacy?

○ Yes

○ No

Q3 **Patient Assessment of Chronic Illness Care (PACIC-11)**

Considering your recent visits to your community pharmacy in the last 3 months to receive care and services for your chronic medical conditions, <u>what percentage of the time were you:</u>

O=None 100=Always

0 10 20 30 40 50 60 70 80 90 100

Given choices about treatment to think about	
Satisfied that your care was well organized	
Helped to set specific goals to improve your eating or exercise	
Given a copy of your treatment plan	
Encouraged to go to a specific group or class to help you cope with your chronic condition	
Asked questions, either directly or on a survey, about your health habits	
Helped to make a treatment plan that you could carryout in your daily life	
Helped to plan ahead so you could take care of your condition even in hard times	
Asked how your chronic condition affects your life	
Contacted after a visit to see how things were going	
Told how visits with other types of doctors, like an eye doctor or surgeon, would help your treatment	
Told how visits with other types of health professionals would help your treatment	
Given enough time to talk about your medical conditions or medications	
Told your pharmacist would work together with other health professionals to coordinate your care	

Q4 Please answer the following statement.

	Extremely satisfied	Moderately satisfied	Somewhat satisfied	Somewhat dissatisfied	Moderately dissatisfied	Extremely dissatisfied
Overall, how satisfied are you with the care you receive by your pharmacist?	○	○	○	○	○	○

The following SIX questions explore your health-related quality of life using a validated tool called the: EQ-5D-5L

Q5 Please click the ONE box that best describes your health TODAY.

MOBILITY

- ○ I have no problems in walking about
- ○ I have slight problems in walking about
- ○ I have moderate problems in walking about
- ○ I have severe problems in walking about
- ○ I am unable to walk about

Copyright © EuroQol Research Foundation. EQ-5D™ is a trade mark of the EuroQol Research Foundation.

Q6 Please click the ONE box that best describes your health TODAY.

SELF-CARE

- ◯ I have no problems washing or dressing myself
- ◯ I have slight problems washing or dressing myself
- ◯ I have moderate problems washing or dressing myself
- ◯ I have severe problems washing or dressing myself
- ◯ I am unable to wash or dress myself

Copyright © EuroQol Research Foundation. EQ-5D™ is a trade mark of the EuroQol Research Foundation.

Q7 Please click the ONE box that best describes your health TODAY.

USUAL ACTIVITIES *(e.g. work, study, housework, family or leisure activities*)

- ◯ I have no problems doing my usual activities
- ◯ I have slight problems doing my usual activities
- ◯ I have moderate problems doing my usual activities
- ◯ I have severe problems doing my usual activities
- ◯ I am unable to do my usual activities

Copyright © EuroQol Research Foundation. EQ-5D™ is a trade mark of the EuroQol Research Foundation.

Q8 Please click the ONE box that best describes your health TODAY.

PAIN / DISCOMFORT

- ◯ I have no pain or discomfort
- ◯ I have slight pain or discomfort
- ◯ I have moderate pain or discomfort
- ◯ I have severe pain or discomfort
- ◯ I have extreme pain or discomfort

Copyright © EuroQol Research Foundation. EQ-5D™ is a trade mark of the EuroQol Research Foundation.

Q9 Please click the ONE box that best describes your health TODAY.

ANXIETY / DEPRESSION

- ◯ I am not anxious or depressed
- ◯ I am slightly anxious or depressed
- ◯ I am moderately anxious or depressed
- ◯ I am severely anxious or depressed
- ◯ I am extremely anxious or depressed

Copyright © EuroQol Research Foundation. EQ-5D™ is a trade mark of the EuroQol Research Foundation.

Q10
We would like to know how good or bad your health is TODAY. The following scale is numbered from 0-100.

100 means the <u>best</u> health you can imagine.
0 means the <u>worst</u> health you can imagine.
Please click on the scale to indicate how your health is TODAY.

	The worst health you can imagine	The best health you can imagine
	0 10 20 30 40 50	60 70 80 90 100

Copyright © EuroQol Research Foundation. EQ-5D™ is a trade mark of the EuroQol Research Foundation.

Q11 (PHQ-2) Over the past 2 weeks, how often have you been bothered by any of the following problems?

	Not at all	Several days	More than half the days	Nearly every day
Little interest or pleasure in doing things	○	○	○	○
Feeling down, depressed or hopeless	○	○	○	○

Copyright © 1999 Pfizer Inc. All rights reserved.

Q12 (GAD-2) Over the last 2 weeks, how often have you been bothered by any of the following problems:

	Not at all	Several days	More than half of the days	Nearly every day
Feeling nervous, anxious, or on edge	○	○	○	○
Not being able to stop or control worrying	○	○	○	○

Q13 (Single Item Literacy Screener) How often do you have someone like a family member, friend, hospital or clinic worker or caregiver help you read health plan materials (such as written information about your health or care you are offered)?

- ○ All of the time
- ○ Most of the time
- ○ Some of the time
- ○ Little of the time
- ○ None of the time

Q14 Please select your sex

- ○ Male
- ○ Female

Q15 Please select your YEAR of birth.

Q16 What is your <u>current</u> marital status?

- ○ Single – never married
- ○ Married/common law
- ○ Separated/Divorce
- ○ Widowed
- ○ Prefer not to respond

Q17 What is the highest level of education you have completed?

- ○ Less than high school
- ○ Completed high school (or equivalent)
- ○ Completed college or technical school (diploma)
- ○ Completed post-secondary training (bachelor’s degree)
- ○ Post-graduate degree (Master’s, PhD, MD)
- ○ Prefer not to respond

Q18 What is your current employment status?

- ○ Employed
- ○ Unemployed
- ○ Retired
- ○ Prefer not to respond

Q19 Which of the following categories best describes your total annual household income?

- ○ Less than $20,000
- ○ $20,000 to $49,999
- ○ $50,000 to $99,999
- ○ More than $100,000
- ○ Prefer not to respond

Q20 Which of the following best describes your ethnicity?

- ◯ Caucasian
- ◯ Aboriginal/Indigenous
- ◯ African
- ◯ Hispanic/Latino
- ◯ Caribbean
- ◯ East Asian
- ◯ South Asian
- ◯ Middle Eastern
- ◯ Prefer not to respond

Q21 Do you have any of the following medical conditions that have been diagnosed by a health professional? (Check all that apply)

- ☐ Asthma
- ☐ Chronic Obstructive Pulmonary Disorder
- ☐ Ischemic Heart Disease
- ☐ Hypertensive Disease
- ☐ Heart Failure
- ☐ Diabetes Mellitus
- ☐ Mental Health Disorder

Thank you for taking the time to complete this survey.

Necyk C, et al. Exploring the impact of pharmacist comprehensive annual care plans on perceived quality of chronic illness care by patients in Alberta, Canada. *Can Pharm J (Ott)* 2021;154. DOI: 10.1177/17151635211020340.
