# Supplementary material for: Exploring the impact of pharmacist comprehensive annual care plans on perceived quality of chronic illness care by patients in Alberta, Canada
Source: Can Pharm J (Ott). 2021 Jul 5;154(5):331–41. doi: 10.1177/17151635211020340 (PMC8408906; doi:10.1177/17151635211020340)
Supplement: sj-pdf-2-cph-10.1177_17151635211020340 – Supplemental material for Exploring the impact of pharmacist comprehensive annual care plans on perceived quality of chronic illness care by patients in Alberta, Canada [file sj-pdf-2-cph-10.1177_17151635211020340.pdf]

APPENDIX 2 Sensitivity Analysis

Demographics of sensitivity analysis subgroup

**Characteristic**<br>*(number of respondents in CACP group; number of respondents in control group)*	**CACP Group (n=79)**	**Control Group (n=192)**	**p-value**
Age (n=68; 153) mean, SD	65 (12)	65 (12)	0.63
Sex (n=74; 171)			0.67
Female	43%	46%	
Male	57%	54%	
Marital Status (n=74; 171)			0.14
Single/never married	10%	11%	
Married/common law	62%	68%	
Separated/Divorced	10%	11%	
Widowed	16%	9%	
Prefer not to respond	3%	2%	
Education Level (n=74; 171)			0.22
Less than high school	5%	6%	
High school	32%	25%	
College/technical school	38%	37%	
Post-secondary	16%	17%	
Post-graduate	15%	13%	
Prefer not to respond	1%	1%	
Annual Income (n=74; 169)			0.44
<$20,000	8%	9%	
$20,000-$49,999	24%	21%	
$50,000-$99,999	31%	31%	
>$100,000	24%	18%	
Prefer not to respond	12%	21%	
Ethnicity (n=74; 169)			0.89
Caucasian	86%	88%	
Aboriginal/Indigenous	1%	0.6%	
African	0%	0%	
Hispanic/Latino	1%	0.6%	
Caribbean	0%	0.6%	
East Asian	1%	1%	
South Asian	3%	3%	
Middle Eastern	0%	0%	
Prefer not to respond	4%	1%	

Qualifying Conditions (n=59;134)			
Asthma	12%	22%	0.09
Chronic obstructive pulmonary disease	20%	13%	0.17
Ischemic heart disease	10%	3%	0.04
Hypertensive disease	51%	40%	0.18
Heart failure	8%	10%	0.67
Diabetes mellitus	31%	36%	0.48
Mental health disorder	22%	15%	0.23

Health and literacy status of sensitivity analysis subgroup

**Survey Question** *(number of respondents in CACP group; number of respondents in control group)*	**CACP Group (n=79)**	**Control Group (n=192)**	**p-value**
EQ-5D-5L Index Value[*] (n=73; 170)	0.77 (0.18)	0.80 (0.15)	0.15
EQ-5D-5L Visual Analogue Scale Score[*] (n=161; 172)	67 (21)	70 (18)	0.30
Single Item Literacy Screener[*] (n=74; 171)	1.6 (1.1)	1.5 (1.0)	0.57
PHQ-2 Score[**] (n=71; 166)			
Equal to or greater than 3	20% (12% to 31%)	13% (9% to 19%)	0.21
GAD-2 Score[**] (n=71; 167)			
Equal to or greater than 3	20% (11% to 31%)	15% (10% to 21%)	0.37

[*]Values reported as mean (SD)
[**]Values reported as proportions (95% CI)

Satisfaction of pharmacy care and care plan awareness of sensitivity analysis subgroup

**Survey Question** *(number of respondents in CACP group; respondents in control group)*	**CACP Group (n=178)**	**Control Group (n=341)**	**p value**
Satisfaction with care from pharmacist[*] (n=73; 174)	4.9 (1.4)	4.9 (1.3)	0.84
Participants reporting they received a care plan[**](n=79; 191)	100%	0	
Participants reporting they signed a care plan[**] (n=77; 190)	62% (51% to 73%)	9% (6% to 15%)	<0.001

[*]Values reported as mean (SD)
[**]Values reported as proportions (95% CI)

Necyk C, et al. Exploring the impact of pharmacist comprehensive annual care plans on perceived quality of chronic illness care by patients in Alberta, Canada. *Can Pharm J (Ott)* 2021;154. DOI: 10.1177/17151635211020340.
